# Longitudinal assessment of age-related changes in antegonial notch depth in untreated individuals

**DOI:** 10.1007/s00784-026-07154-6

**Published:** 2026-10-17

**Authors:** Mohamad Rustom, Balazs J. Denes, Stavros Kiliaridis, Gregory S. Antonarakis

**Affiliations:** 1 Division of Orthodontics, University Clinics of Dental Medicine, University of Geneva, 1 rue Michel-Servet, Genève, 1206 Switzerland; 2 Department of Orthodontics and Dentofacial Orthopedics, University of Bern, Freiburgstrasse 7, Bern, 3010 Switzerland

**Keywords:** Antegonial notch, Mandibular growth, Cephalometry, Craniofacial development, Multilevel modelling, Orthodontics

## Abstract

**Introduction:**

The role of the antegonial notch as an indicator of growth remains controversial in the literature. This study examined how antegonial notch depth changes during growth and whether craniofacial morphology is associated with its depth.

**Methods:**

A longitudinal sample of 250 untreated healthy individuals (42% female) from the American Association of Orthodontists Foundation Craniofacial Growth Legacy Collection was analysed at three time points: ages 7–8, 13–14, and 17–18 years. Antegonial notch depth was measured from standardized lateral cephalograms. Multilevel general linear regression was applied to evaluate whether skeletal morphology could predict antegonial notch depth.

**Results:**

Antegonial notch depth changed significantly over time (*p* < 0.001) with a medium effect size. Males consistently had deeper notches than females. Sagittal molar relationships showed no significant influence on notch depth. Multilevel modelling revealed that only the gonial angle was a statistically significant predictor of notch depth (B = − 0.014, *p* = 0.044), with deeper antegonial notches being measured in individuals with smaller gonial angles, while the intermaxillary angle showed no significant association (*p* = 0.064). Neither ANB nor the mandibular plane angle had any significant predictive value on antegonial notch depth.

**Conclusion:**

Antegonial notch depth undergoes modest sex-specific changes during adolescence and is minimally associated with craniofacial morphology. Only the gonial angle was a significant predictor (deeper notches in mandibles with smaller gonial angles), suggesting that notch depth primarily reflects localized bone remodelling.

## Introduction

Accurate prediction of mandibular growth could be an important element to orthodontic and orthognathic treatment planning, enabling timely and effective interventions in developing patients. One anatomical feature of interest is the antegonial notch (also known as the gonial notch, premasseteric notch, or groove for facial vessels), a concavity on the inferior border of the mandible, located anterior to the masseter and medial pterygoid attachments [1–3]. Its prominence can range from absent to deep, with mean depths being around 2.0 ± 1.1 mm for orthodontically untreated individuals under the age of 18 in North America [4, 5].

Mechanistic theories suggest notch formation results from differential muscular activity. Wolf’s law states that the bone remodels itself based on the load to reach an optimal shape [6, 7]. Recent evidence however showed weak correlation between antegonial notch dimensions and skeletal parameters but no association with masticatory muscle size [7, 8]. On the other hand, Hovell proposed that continued masseter and medial pterygoid activity promotes bone apposition at the gonial angle and notch development, whereas balanced mandibular and soft-tissue growth yields an absent notch.

It is important to note that the antegonial notch depth and presence seems to differ depending on ethnicity. Obamiyi et al. showed that those with African, Asian and Hispanic ethnicities tend to present with an antegonial notch more often than the White group [9]. As for the depth, a study on a Japanese population showed that the mean depth was 3.1 ± 1.1 mm which was more than the North American group [10], but this may also be dependent on age. Sex seems to be of relevance with males presenting with a significantly deeper notch [5, 11]. Such sexual dimorphism is consistent with broader mandibular shape variation between males and females reported in recent morphometric analyses [11].

Schütz et al. conducted a retrospective analysis of the antegonial notch depth using a sample of growing children (*n* = 302) from North America [5]. The results showed that males have significantly deeper notches than females (1.2 mm difference). The depth of the notch in those with Class I occlusion was significantly higher than for those with Class II malocclusions. For those with a deep notch (which all happened to be male in the study), the changes occurred largely during or after the adolescent growth spurt between the ages of 13 and 18. Nonetheless, a shortcoming of the Schütz et al. [5] study was that it does not adequatly take into account the multilevel structure of the paired data –time points should be nested within participants – therefore, statistical tests and comparisons could be inaccurate due to the violation of the independence of observation assumption [12].

Despite these anatomical insights, evidence for clinical utility remains conflicting. Björk’s longitudinal implant studies indicated a correlation between notch depth and mandibular rotation direction—deep notches with vertical growth, shallow with horizontal [1, 13, 14]. Singer et al. and Lambrechts et al. similarly reported that deep notches correspond to increased vertical facial growth, increased facial height, and obtuse gonial angles [15, 16]. On the other hand, however, Halazonetis et al. and Kolodziej et al. countered that notch depth shows weak or no predictive value for mandibular rotation or future facial growth [17, 18]. Moreover, Kolodziej et al. in their longitudinal study of untreated normal subjects concluded that while a statistically significant relationship exists between notch depth and horizontal jaw growth, the correlation might lack clinical significance [18].

Thus, although the antegonial notch remains a promising anatomical marker, its relevance in routine growth prediction—especially in normative dental populations—remains unresolved, and longitudinal investigations in untreated cohorts are needed. The present study contributes to the field by using a longitudinal untreated cohort assessed at three developmental stages and by applying multilevel modelling to better reflect the hierarchical structure of the data. Its novelty lies in examining normal developmental changes in antegonial notch depth while simultaneously testing whether common cephalometric variables can predict it.

The present longitudinal study evaluated orthodontically untreated participants to (1) assess the development of the antegonial notch during normal growth; and (2) determine whether cephalometric craniofacial morphology is associated with and can predict antegonial notch depth. This objective was intended to test the potential clinical utility of antegonial notch depth as a marker for monitoring mandibular growth in general populations. The study hypothesis was that antegonial notch depth changes significantly during growth and differs according to sex and sagittal molar relationships.

## Materials and methods

### Sample selection

For this retrospective study, lateral cephalograms of 250 healthy individuals of which 42.4% (*n* = 106) were female, were collected from the American Association of Orthodontists Foundation (AAOF) Craniofacial Growth Legacy Collection website (www.aaoflegacycollection.org). Formal ethical approval and additional informed consent were not required, as the raw data (lateral cephalograms) are publicly accessible through the stated resource. The study was conducted in compliance with the Declaration of Helsinki. Records were taken from all nine growth studies available on the online database, including all three dental sagittal relationships according to Angle (Class I, Class II and Class III), where three lateral cephalometric radiographs were available at the ages of 7–8 years (T1), 13–14 years (T2) and 17–18 years (T3) for the each participant. The majority of participants had Class I (67.2%, *n* = 168) relationships, 29.6% (*n* = 74) had a Class II malocclusion, and 3.2% (*n* = 8) were Class III. These three age groups represent preadolescent children, adolescents, and young adults [18]. The ethnicity for our sample was either White or African American. Exclusion criteria were: previous orthodontic treatment, missing radiographs at any of the three time points, incomplete measurements, or missing key study variables. Regarding the time frame of radiograph acquisition, these records originate from the AAOF Craniofacial Growth Legacy Collection, implying that the images were historically acquired as part of the original longitudinal growth studies included in this database; overall the longitudinal dataset radiographs were collected between 1935 and 1985 from across the US. and Canada.

### Data collection and analysis

**Fig. 1:**
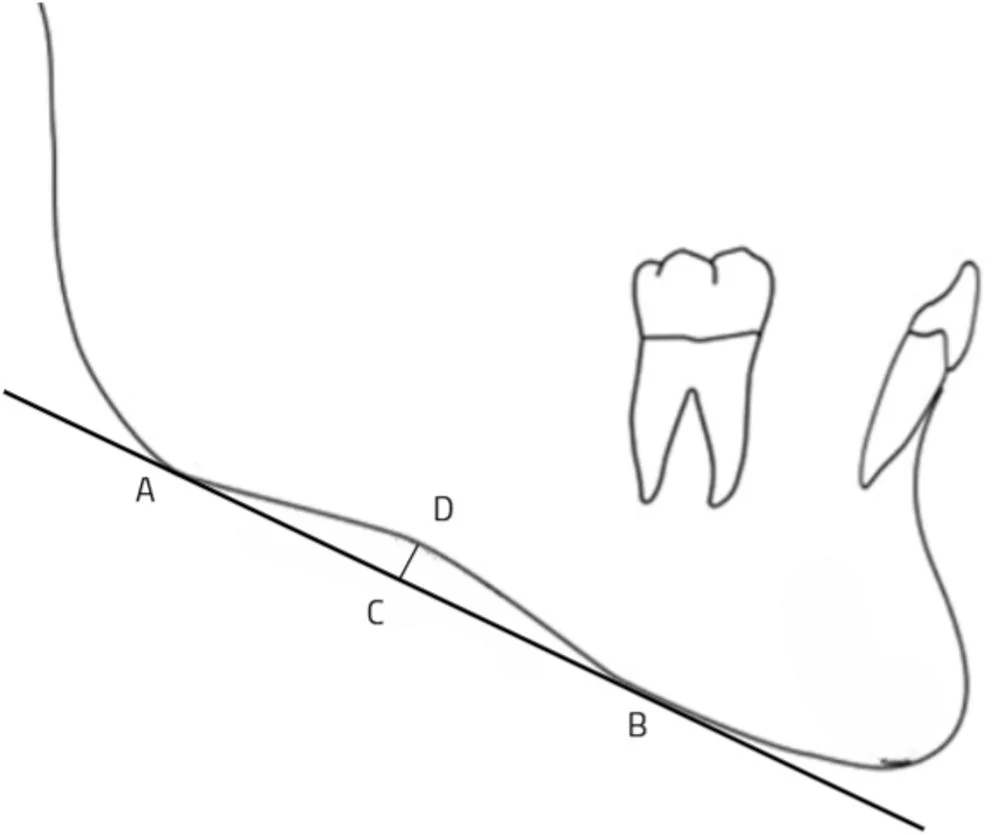
Lateral cephalometric tracing representing antegonial notch depth. Notch depth was measured as the perpendicular distance between point D (representing the deepest point of the antegonial concavity) and point C (perpendicular dropped from D onto the mandibular line (ML), defined by the tangent to the inferior border of the mandible using points A and B as the anterior and posterior points of contact of the tangent)

The lateral cephalograms were calibrated, traced and analysed by a single operator (M.R.), using Osirix software (OsiriX v3.9 Pixmeo SARL). It should be noted that lateral cephalograms inherently superimpose the right and left craniofacial structures, preventing side-specific differentiation. The magnification criteria were provided on the AAOF Craniofacial Growth Legacy Collection website for each growth study group in order to correctly calibrate all radiographs and accurately measure both linear and angular values on the radiographs. Antegonial notch depth was selected as the primary outcome because it represents the most established and straightforward cephalometric measure of the notch in the orthodontic literature. In contrast, length and area require broader contour delineation and are likely to be more affected by the two-dimensional nature of lateral cephalograms, including projection distortion and superimposition of bilateral structures. The antegonial notch was defined as the bony concavity located on the inferior border of the mandible, anterior to the masseteric attachment, consistent with established anatomical descriptions of this mandibular landmark. The deepest point of this concavity was identified as point D (Fig. 1). The mandibular line (ML) was constructed as the tangent to the inferior border of the mandible using points A and B, and point C was defined as the point on this tangent intersected by the perpendicular dropped from point D. Antegonial notch depth was then measured as the perpendicular distance from point D to point C on the ML line (Fig. 1). In case of double contours, for example where the border of the right and left mandibular bodies did not coincide, points and lines were traced as a mean distance between right and left contours. This was measured on the lateral cephalometric radiographs at all three ages for each individual.

Craniofacial morphology was assessed on the lateral cephalometric radiographs using standard cephalometric landmarks, including: Sella (S), Nasion (N); Anterior Nasal Spine (ANS); Posterior Nasal Spine (PNS); Point B, the point of maximum concavity on the anterior mandible; Articulare (Ar); Gonion (Go); and Menton (Me). From these landmarks, the following angular measurements were obtained: ANB, mandibular plane angle (SN-MeGo), intermaxillary angle (ANSPNS-MeGo), and gonial angle (Ar-Go-Me).

### Error of the method

To evaluate both systematic and random errors in localizing landmarks, 30 lateral cephalograms were randomly chosen (using the true random number generator - https://www.random.org) and retraced by the same examiner. Systematic error was calculated using paired t-tests, revealing no significant systematic error (*p* > 0.05). Random error was assessed using Dahlberg’s formula (based on the root mean square of the differences between the duplicate tracings divided by two, yielding results in the original unit of measurement), and the maximum error was found to be 0.3 mm for linear and 1.1° for angular measurements.

### Statistical analysis

Statistical analyses were conducted using SPSS (IBM SPSS Statistics for Windows, version 29.0; IBM Corp., Armonk, NY, USA, 2022). Data normality checks were conducted for continuous variables using the skewness (-2, + 2) and Kurtosis (-7, + 7) and variables were within acceptable limits. There were very few outliers in the entire dataset. Removal of outliers identified using the 1.5 × IQR criterion had only a minor effect on means and standard deviations. Therefore, outliers were included in the analysis and were not deleted.

Descriptive statistics were calculated for antegonial notch depth at each age according to sex and Angle classification. Longitudinal differences in antegonial notch depth were first examined using a mixed repeated-measures ANOVA, with time as the within-subject factor and sex and Angle classification as fixed between-subject factors. Where the sphericity assumption was not met, Greenhouse–Geisser correction was applied. Since repeated measurements were clustered within individuals, the intraclass correlation coefficient (ICC) was calculated to quantify within-subject dependency. A two-sided *p* value of less than 0.05 was considered statistically significant. A multilevel general linear mixed model was fitted to examine changes in antegonial notch depth over time and its association with craniofacial morphology, while accounting for repeated observations within participants. Antegonial notch depth was entered as the dependent variable. Time, ANB angle, mandibular plane angle, intermaxillary angle, and gonial angle were included as fixed effects. Participant-level random effects were specified to allow both the intercept and the slope for time to vary across individuals. The multilevel model can be written as:

$$\begin{array}{c}AND_{ij}\beta_0+\beta_1\left(Time_{ij}\right)+\beta_2\left(AND_{ij}\right)\\+\beta_3\left(MPA_{ij}\right)+\beta_4\left(IMA_{ij}\right)+\beta_5\\\left(GA_{ij}\right)+u_{0i}+u_{1i}\left(Time_{ij}\right)+\varepsilon_{ij}\end{array}$$

Where:

- $AND_{ij}$ = antegonial notch depth for participant i at time point j
- $$\beta_0$$ = fixed intercept
- $\beta_1+\beta_5$ fixed-effect coefficients
- *Time* = age.
- *ANB* = ANB angle
- *MPA* = mandibular plane angle
- *IMA* = intermaxillary angle
- *GA* = gonial angle
- $u\left\{0i\right\}$ = random intercept for participant i
- $u\left\{1i\right\}$ = random slope for time for participant i
- $$\in_{ij}$$ = residual error

Repeated-measures ANOVA was used to test whether antegonial notch depth differed significantly across time and between the studied groups. Multilevel modelling was used to examine whether antegonial notch depth could be predicted by selected craniofacial morphology variables while accounting for the clustering of repeated measurements within individuals.

To assess the robustness of the pooled data against potential source-study effects prior to longitudinal modeling, a two-part sensitivity analysis was conducted. First, a one-way analysis of variance was used to evaluate baseline heterogeneity among the contributing study cohorts for each cephalometric variable. Subsequently, a leave-one-out sensitivity analysis was performed to quantify the influence of individual datasets on the aggregate estimates. This process involved iteratively recalculating the overall pooled means while systematically excluding one study at a time to ensure that no single data source biased the statistical outcomes of the combined sample. Because no single study skews the aggregate data, it was concluded that pooling the data for the longitudinal analysis is methodologically sound.

## Results

**Fig. 2:**
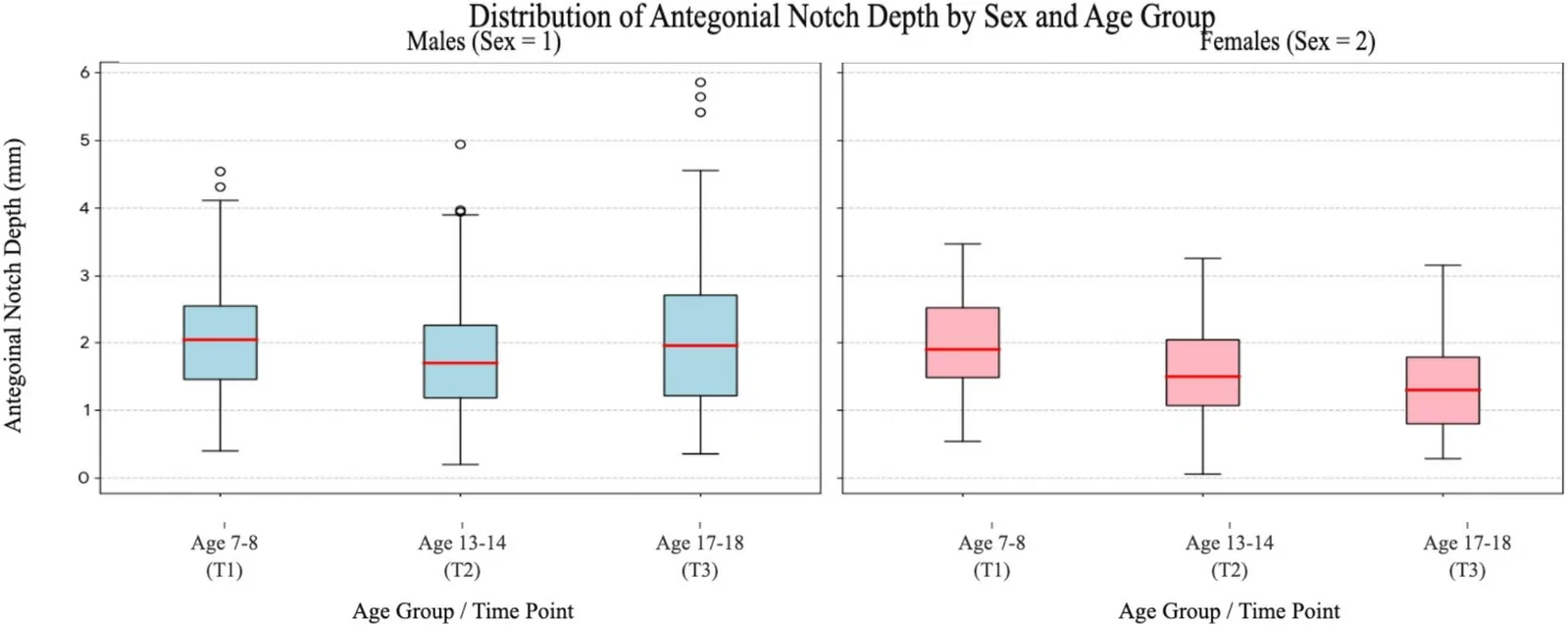
Sex-specific longitudinal distribution of antegonial notch depth. Box plots display the median (red horizontal line), interquartile range (box), and range (whiskers) at T1 (7–8 yrs), T2 (13–14 yrs), and T3 (17–18 yrs). Individual points represent outliers. Antegonial notch depth differed significantly across time points (repeated-measures ANOVA, *p* < 0.001)

Descriptive statistics (Table 1) revealed that on average males exhibited deeper antegonial notches than females at all time points, with the greatest divergence observed at age 18, where males had a mean depth of 2.10 mm (SD = 1.10) compared to 1.39 mm (SD = 0.65) in females. Within time comparison for each sex subgroup, it could be noticed that the male group notch depth fluctuated dropping at 14 years of age then increasing at 18 years in contrast to the female group which consistently decreased at each time point (Fig. 2).

**Table 1:** Findings of mixed repeated measures ANOVA for the antegonial notch depth for the three time points across sex and occlusal class subgroups

Time point	Sex		Angle class			Effects		
	Male (*n* = 144)	Female (*n* = 106)	Class I (*n* = 168)	Class II (*n* = 74)	Class III (*n* = 8)		*p*	ES (η_*p*_²)
	mean ± SD	mean ± SD	mean ± SD	mean ± SD	mean ± SD	Time	< 0.001	0.037
T1 (yr 7–8)	2.05 ± 0.78	1.96 ± 0.68	2.09 ± 0.78	1.82 ± 0.63	1.98 ± 0.66	Time × Sex	0.001	0.029
T2 (yr 13–14)	1.76 ± 0.86	1.57 ± 0.71	1.72 ± 0.83	1.58 ± 0.72	1.67 ± 1.01	Time × Class	0.448	0.007
T3 (yr 17–18)	2.10 ± 1.10	1.39 ± 0.65	1.88 ± 1.04	1.64 ± 0.86	1.57 ± 1.16	Time × Sex × Class	0.709	0.004

The unit of measurement for the antegonial notch depth is millimetres

*SD* standard deviation, *n* number of participants, *p* probability that the difference is due to chance, *ES* effect size, *ηp²* partial eta squared

Repeated measures ANOVA showed that antegonial notch depth significantly fluctuated between the three time points measured (*p* < 0.001) with a medium effect size (η_p_²=0.09) according to Cohen 1988 [19]. Post-hoc tests showed that all time points differed significantly from one another.

Per class comparison shows that antegonial notch depth fluctuated for Class I (female = 41.7%) and II (female = 44.6%) sagittal relationships, decreasing at 13–14 years of age, then slightly increasing at 17–18 years (Table 1). It is noteworthy that findings related to Class III need to be interpreted with caution due to the small sample size.

### Changes in antegonial notch depth during growth

A mixed repeated measures ANOVA was conducted to evaluate changes in antegonial notch depth across three developmental time points (ages 7–8, 13–14, and 17–18 years) accounting for sex and sagittal relatopnships as fixed factors (Table 1). The analysis showed a significant main effect of time (*p* < 0.001, partial η² = 0.037), indicating that antegonial notch depth varied significantly over time with a small effect size according to Cohen 1988 [19]. The time and sex interaction were also statistically significant with a small effect size (*p* = 0.001, partial η² = 0.029), suggesting that the pattern in notch depth across time differed meaningfully between males and females (Table 1). The time and molar class interactions, and the time and molar class and sex interactions were not statistically significant (*p* > 0.05).

### Craniofacial morphology as a predictor of antegonial notch depth

Interclass Correlation Coefficient (ICC) was 0.63 showing a clear need for multilevel modelling. The multilevel model (Table 2) shows that none of the studied craniofacial morphologies significantly predict antegonial notch depth except for the gonial angle (B=-0.014, *p* = 0.04), but with a small effect size (ES = 0.20). The intermaxillary angle (ANSPNS – ML) was not a statistically significant predictor (B=–0.0191, *p* = 0.064). The model shows that gonial angle values were associated with a shallower antegonial notch (negative correlation).

**Table 2:** Multilevel general linear model of participants’ antegonial notch depth as predicted by different craniofacial morphologies

Effect type	Predictor	B	SE	*p*	ES
Fixed	Intercept	3.9627			
	Time	–0.1240	0.0297	< 0.001	0.39
	ANB	0.0165	0.0154	0.283	0.10
	Mandibular plane angle	0.0091	0.0111	0.415	0.08
	Intermaxillary angle	–0.0191	0.0103	0.064	0.18
	Gonial angle	–0.0140	0.0069	0.044	0.20
Random	Intercept	0.4086			
	Time	0.0663			
	Residual	0.1900			

*B* unstandardized regression estimate, *SE* standard error, *p* probability that the association or the difference is due to chance, *ES* Effect size

## Discussion

The present study aimed to assess the development of the antegonial notch during growth in orthodontically-untreated subjects and to determine whether cephalometric craniofacial morphology measures can predict the depth of the antegonial notch. In this longitudinal cohort of untreated individuals, antegonial notch depth showed statistically significant changes over time with a medium effect size. Males consistently exhibited deeper notches than females at all ages [11]. Both sexes showed a decrease in notch depth from ages 7–8 to 13–14, but thereafter males experienced an increase by age 17–18 while female notch depths continued to become more shallow. Sagittal molar relationships (Angle class I, II, III) showed no significant main effect on antegonial notch development, although the small Class III subgroup limits any inference for this group. Although Angle class is clinically used as a proxy for dentofacial pattern, its sagittal dental categorization may not capture the vertical/rotational and functional determinants of inferior-border remodelling that may influence antegonial notch depth. Multilevel modelling showed that only the gonial angle could significantly predict notch depth, with larger gonial angles associated with slightly shallower notches, although the clinical significance of this finding is questionable due to the small effect size.

The pronounced sex difference and timing of change hint at underlying mechanisms. Males not only had deeper notches on average, but their notches deepened after age 13–14, whereas females’ notches continued to flatten, as also suggested by other data [11]. The most defensible biological interpretation is that antegonial notch depth reflects local mandibular remodelling at the inferior border near the gonial/antegonial region, which is sensitive to (i) growth-related rotation and remodelling and (ii) sexually dimorphic timing and intensity of the pubertal spurt (mandibular growth and muscle forces increase later and more in males). This observation aligns with broader morphometric evidence that mandibular shape is highly sexually dimorphic beyond notch morphology, which is in line with previous results [11]. This pattern is consistent with muscle-driven remodelling suggested in the literature [6, 20, 21]. In puberty, male children typically experience a surge in masticatory muscle mass, which could stimulate bone apposition at the gonial angle and accentuate the notch [5, 7]. Indeed, Schütz et al. interpreted their results with this in mind, suggesting that increased notch depth in males may be due to stronger masticatory forces and greater muscle mass [5]. Our finding suggest that notch depth in healthy individuals correlates negatively with the gonial angle (i.e. deeper notches in mandibles with smaller gonial angles). Although this negative association is statistically significant, its effect size was small and may partly be explained by shared anatomical features, the proximity of cephalometric landmarks, or local remodeling phenomena, rather than a strong independent predictive relationship [7].

Clinically, these findings suggest caution in interpreting antegonial notch depth as a marker of craniofacial morphology, as the associations observed were somewhat weak. Although statistically significant, the effects we observed are small to medium. Thus, antegonial notch depth may not be a robust standalone marker for treatment timing or growth forecasts. It may instead serve as an indicator of individual mandibular morphology or muscle function. The absence of strong correlations with conventional cephalometric angles (such as the ANB or the mandibular plane angle) suggests that notch depth is more a feature of local bone remodelling. This aligns with the view that the antegonial notch should not be overinterpreted as a “growth potential” gauge [7].

Our results are partly consistent with, and partly differ from, previous studies. Consistent with Schütz et al., we found that males have deeper notches than females and that most deepening occurs around puberty [5], keeping in mind that part of the material between the two studies is common since it is derived from the AAOF Craniofacial Growth Legacy Collection. However, unlike Lambrechts et al. and Singer et al. ^15,16^ who proposed deep notches as markers of a hyperdivergent (backward-rotating) growth pattern, we observed the opposite tendency. In our sample of healthy individuals, deeper notches were linked to smaller gonial angles (perhaps related to a more horizontal growth orientation). This agrees more closely with Kolodziej et al., who reported that antegonial notch depth is not a reliable indicator of growth direction [18]. For example, whereas Lambrechts et al. and Singer et al. found deep notches in vertical growers, Kolodziej et al. found no clinically useful correlation between notch depth and future mandibular growth. Similarly, Halazonetis and co-workers also noted only weak or non-significant relationships between notch depth and craniofacial angles [17]. Overall, earlier growth-prediction studies tended to associate deeper notches with larger gonial angles and vertical growth patterns. It should also be noted that our analysis found no significant association with the mandibular plane angle, and other vertical parameters were not assessed, so comparisons with studies of vertical growth should be made with caution. However, more recent or methodologically cautious studies suggest that the correlation is weak, inconsistent, or clinically limited. Thus, antegonial notch depth may not be a robust standalone marker for treatment timing or growth forecasts. It may instead reflect individual mandibular morphology and, possibly, aspects of muscle-related functional loading, although evidence for such a relationship remains mixed [7, 10, 22].

Notably, Schütz et al. reported only a fractional average difference (≈ 0.33 mm) between antegonial notches in individuals with Class I and II malocclusions, cautioning that this is about the same magnitude as the measurement error [5]. We observed the same: any differences in notch depth between individuals with different Angle molar relationships were insignificant and within a 0.5 mm threshold. This suggests that sagittal occlusion (in orthodontically-untreated individuals) has minimal influence on notch depth during growth. The consistency between our results and those of Schütz et al. [5] particularly regarding sex differences and the timing of notch change strengthens confidence in these findings despite the sample being derived from the same source (namely the AAOF).

### Limitations

The present study has certain limitations. First, the antegonial notch was not normalized to mandibular size. It is possible that the observed differences related to sex and growth may simply reflect general variations in jaw dimensions as larger mandibles naturally tend to exhibit larger notches. Another key limitation is that the magnitude of statistical differences between groups could possibly be within the measurement error and therefore may lack clinical relevance. This concern is strengthened by the use of lateral cephalometric radiographs, where superimposition of bilateral structures can reduce measurement accuracy and obscure precise identification of the antegonial notch. In addition, the inherent anatomical asymmetry of the antegonial notch was not explicitly accounted for due to the superimposition. This also was a retrospective study, relying on historical growth-study radiographs with variable quality. Although we calibrated images, older film images can introduce noise. In addition, all measurements were performed by a single examiner, so inter-examiner reliability could not be assessed, which may affect measurement reproducibility. Our sample, drawn from predominantly White or African-American cohorts, limits generalization to other ethnic groups. The relatively small number of individuals with Class III malocclusion reduces power to detect differences in these individuals. Importantly, we used mixed (multilevel) modelling to account for repeated measures, addressing a key shortfall of past analyses which violated the assumption of independence of observation. Future studies could improve on this by using 3D imaging to capture notch morphology more fully, and by directly measuring muscle size or function [3]. Longitudinal research in larger and more diverse samples is needed to confirm these developmental findings.

## Conclusion

Antegonial notch depth changes only modestly during normal growth in healthy individuals, while being consistently deeper in males. It seems to be minimally associated with craniofacial morphology, with only the gonial angle showing up as a significant predictor (deeper notches in mandibles with smaller gonial angles). This suggests that notch depth primarily reflects localized bone remodelling. 

## Data Availability

All raw data are publicly available on the American Association of Orthodontists Foundation (AAOF) Craniofacial Growth Legacy Collection website (https://www.aaoflegacycollection.org).
